# RETRACTION: Identification of Kinesin Family Member 2A (KIF2A) as a Promising Therapeutic Target for Osteosarcoma

**DOI:** 10.1155/bmri/9808573

**Published:** 2025-06-12

**Authors:** BioMed Research International

RETRACTION: Z-X. Wang, S-C. Ren, Z-S. Chang, and J. Ren, “Identification of Kinesin Family Member 2A (KIF2A) as a Promising Therapeutic Target for Osteosarcoma,” *BioMed Research International*, 2020 (2020): 7102757, https://doi.org/10.1155/2020/7102757.

The above article, published online on 3 November 2020 in Wiley Online Library (wileyonlinelibrary.com), has been retracted by agreement between the Section Editor of the journal, Gerald Brandacher; and John Wiley & Sons Ltd.

The retraction has been agreed following an investigation of the concerns raised by *Hoya camphorifolia* on PubPeer [1], which identified multiple instances of inappropriately overlapping figures.

Specifically:

− Figure 2b/d: The Western Blot bands for KIF2A and *β*-actin are identical to the bands corresponding to LAPTM4B and *β*-actin in Figure 2b of [2].
− Figure 3a The image of the cell cultures and the number of colonies presented in the figure are identical to Figure 3b in [2], with the same colonies depicted in both figures (MG-63 and U2OS).
− Figure 3c The 2 bottom images of the wound closure assay have been duplicated in Figure 3(c) of [3], the top left panel of which has also been spotted in Figure 3(c) of [4] (bottom-left panel).
− Figure 3d The immunohistochemistry images have been fully duplicated in Figure 3b of [2], while two panels were identified in Figure 3(d) of [5].

As a result of the investigation, the data and conclusions of this article are considered unreliable.

The authors have been informed of the decision to retract the article but did not provide a response.
